# Assessment of hepatic fibrosis and inflammation with look-locker T1 mapping and magnetic resonance elastography with histopathology as reference standard

**DOI:** 10.1007/s00261-022-03647-6

**Published:** 2022-08-29

**Authors:** Sophie von Ulmenstein, Sanja Bogdanovic, Hanna Honcharova-Biletska, Sena Blümel, Ansgar R. Deibel, Daniel Segna, Christoph Jüngst, Achim Weber, Thomas Kuntzen, Christoph Gubler, Cäcilia S. Reiner

**Affiliations:** 1 Diagnostic and Interventional Radiology, University Hospital Zurich, Raemistrasse 100, 8091 Zurich, Switzerland; 2 Diagnostic Radiology, Balgrist University Hospital, Zurich, Switzerland; 3 Department of Pathology and Molecular Pathology, University Hospital Zurich, Zurich, Switzerland; 4 Division of Gastroenterology and Hepatology, University Hospital Zurich, Zurich, Switzerland; 5 Department of Visceral Surgery and Medicine, Inselspital, Bern University Hospital, University of Bern, Bern, Switzerland; 6 Gastroenterology and Hepatology, Kantonsspital Aarau, Aarau, Switzerland; 7 Gastroenterology and Hepatology, Stadtspital Triemli, Zurich, Switzerland

**Keywords:** MR elastography, Fibrosis, T1 mapping, Liver, Biopsy

## Abstract

**Purpose:**

To compare the diagnostic performance of T1 mapping and MR elastography (MRE) for staging of hepatic fibrosis and grading inflammation with histopathology as standard of reference.

**Methods:**

68 patients with various liver diseases undergoing liver biopsy for suspected fibrosis or with an established diagnosis of cirrhosis prospectively underwent look-locker inversion recovery T1 mapping and MRE. T1 relaxation time and liver stiffness (LS) were measured by two readers. Hepatic fibrosis and inflammation were histopathologically staged according to a standardized fibrosis (F0–F4) and inflammation (A0–A2) score. For statistical analysis, independent *t* test, and Mann–Whitney *U* test and ROC analysis were performed, the latter to determine the performance of T1 mapping and MRE for fibrosis staging and inflammation grading, as compared to histopathology.

**Results:**

Histopathological analysis diagnosed 9 patients with F0 (13.2%), 21 with F1 (30.9%), 11 with F2 (16.2%), 10 with F3 (14.7%), and 17 with F4 (25.0%). Both T1 mapping and MRE showed significantly higher values for patients with significant fibrosis (F0-1 vs. F2-4; T1 mapping *p* < 0.0001, MRE *p* < 0.0001) as well as for patients with severe fibrosis or cirrhosis (F0-2 vs. F3-4; T1 mapping *p* < 0.0001, MRE *p* < 0.0001). T1 values and MRE LS were significantly higher in patients with inflammation (A0 vs. A1-2, both *p* = 0.01). T1 mapping showed a tendency toward lower diagnostic performance without statistical significance for significant fibrosis (F2-4) (AUC 0.79 vs. 0.91, *p* = 0.06) and with a significant difference compared to MRE for severe fibrosis (F3-4) (AUC 0.79 vs. 0.94, *p* = 0.03). For both T1 mapping and MRE, diagnostic performance for diagnosing hepatic inflammation (A1-2) was low (AUC 0.72 vs. 0.71, respectively).

**Conclusion:**

T1 mapping is able to diagnose hepatic fibrosis, however, with a tendency toward lower diagnostic performance compared to MRE and thus may be used as an alternative to MRE for diagnosing hepatic fibrosis, whenever MRE is not available or likely to fail due to intrinsic factors of the patient. Both T1 mapping and MRE are probably not sufficient as standalone methods to diagnose hepatic inflammation with relatively low diagnostic accuracy.

## Introduction

The evaluation and early diagnosis of hepatic fibrosis are important for treatment of patients with chronic liver disease, ideally to prevent the transformation into cirrhosis and thereby decreasing the risk of hepatocellular carcinoma [1, 2] and progression to end-stage liver disease. To avoid a liver biopsy, non-invasive methods are usually preferred by patients. Furthermore, non-invasive imaging methods can give an overview of the whole liver, while liver biopsy is prone to sampling error especially in heterogeneous liver disease [3, 4].

Magnetic resonance elastography (MRE) has been evaluated in multiple studies as a non-invasive method for staging hepatic fibrosis and has been proven valid with high inter- and intra-observer reproducibility and diagnostic performance for the staging of hepatic fibrosis [5, 6]. However, this method has some technical limitations in patients with ascites, iron deposition, and high body mass index [7]. Depending on the magnetic field strength and the available MRE sequence, failure rates between 1.9% and 19.3% have been reported [7, 8]. Furthermore, for MRE hard- and software needs to be available as well as technical know-how [9]. Furthermore, the diagnostic performance of MRE for staging fibrosis can be influenced by hepatic inflammation often coexisting with fibrosis [10].

Exploring alternative, reproducible, cross-sectional imaging methods, recent studies proposed MR relaxometry with T1 mapping as an alternative method to assess hepatic fibrosis [11–14]. The T1 relaxation time of tissue is influenced by the molecular environment of water molecules in the tissue. The excessive accumulation of extracellular matrix with an increase in the collagen volume fraction leads to an increase of T1 relaxation time as shown in myocardial fibrosis [15]. Similarly, increased T1 relaxation times have been shown to correspond with increased hepatic fibrosis [11, 16]. An advantage of T1 mapping is its robustness in patients with high BMI or ascites [17], and it does not need any additional hardware. T1 relaxation times may also be influenced by hepatic inflammation as shown a recent study on patients with acute liver disease [13].

To date, there is no other study with histopathology as standard of reference in patients with mixed etiologies of chronic liver disease evaluating whether T1 mapping shows similar diagnostic performance as MRE for staging hepatic fibrosis and may serve as an alternative to MRE. Also the role of MRE and T1 mapping in grading hepatic inflammation has not been established yet.

Therefore, the purpose of this study is to compare the diagnostic performance of T1 mapping and MRE for staging of hepatic fibrosis with histopathology as standard of reference.

## Materials and methods

This single-center prospective cohort study was approved by the local ethics committee and written informed consent was obtained from all participants.

### Participants

In this prospective cohort study, patients undergoing liver biopsy for suspected diffuse non-malignant liver diseases or exclusion of liver disease in potential living liver donors were recruited between August 2015 and March 2020 to undergo a study MRI within 4 weeks after liver biopsy. In addition, patients with an established diagnosis of cirrhosis induced by alcoholic liver disease (ALD), non-alcoholic fatty liver disease (NAFLD), or hepatitis C undergoing screening MRI for hepatocellular carcinoma were recruited between November 2018 and March 2020. In these patients the diagnosis of cirrhosis was based on previous liver biopsies or clinical, radiographic, and serological findings. Exclusion criteria were (1) general contraindications for MRI, (2) pregnant patients, (3) patients with hepatic iron overload, either as a result in the biopsy (iron > 16 mmol/kg) or on the R2* images with values > 100 s^−1^, (4) severe artifacts on MRE or T1 maps, and (5) proton density fat fraction (PDFF) > 15%.

### Histopathology

The formalin-fixed, paraffin-embedded liver tissue samples were stained with hematoxylin–eosin and Sirius red staining. Liver biopsy samples (*n* = 57) were pathomorphologically evaluated by METAVIR, NAS, or Batts–Ludwig score where appropriate [18, 19]. Both fibrosis stage (staging) and activity of inflammation (grading) were evaluated and then standardized to the in-house-developed common scale (staging: F 0, 1, 2, 3, 4; grading: A 0, 1, 2, 3) (Table 1).

**Table 1:** Unification of histopathological fibrosis staging and activity grading systems [18, 19]

Fibrosis staging Unified scale	METAVIR	Staging of steatohepatitis	Batts–Ludwig
0	F0	0	0
1	F1	1a, 1b	1
2	F2	1c, 2	2
3	F3	3	3
4	F4	4	4

Activity grading Unified scale	METAVIR	NAS score	Batts–Ludwig
0	A0	0–2	0
1	A1	3–4	1, 2
2	A2	5–6	3
3	A3	7–8	4

Fibrosis staging and activity grading systems were used according to the underlying liver disease

### MRI protocol

MRI images were acquired on a 3 Tesla MR system (Magnetom Skyra, Siemens Healthineers, Erlangen, Germany) with a 60-channel body coil. T2-weighted single shot fast spin echo sequences were performed in coronal (without fat saturation) and axial plane (with fat saturation) for anatomical correlation. An axial 3D multi-gradient-echo sequence was acquired with multi-step adaptive fitting algorithm reconstruction (Siemens LiverLab) to derive PDFF and R2* maps (repetition time (TR) 9.0 ms; echo time (TE) 1.05, 2.46, 3.69, 4.92, 6.15, 7.38 ms; flip angle, 4°; field of view (FOV) 450 mm; matrix 160 × 111; ST 3.5 mm; voxel size 1.4 × 1.4 × 3.5 mm).

For T1 mapping, a Look-Locker 2D gradient-echo sequence with inversion recovery pulse was acquired in a single breath-hold (TR 3.00 ms, TE 1.32 ms, flip angle 8°, 8-mm slice thickness, FOV 380 mm, matrix of 380 × 309, and scan time of 18 s). For the MRE, an echoplanar imaging (EPI) sequence (TR 1400 ms, TE 47 ms, 6-mm slice thickness, FOV 384 mm, matrix 384 × 384, and scan time 15 s) was used. To perform the MRE, a resoundant (Resoundant Inc., Rochester, MN, USA) was positioned on the right upper abdomen creating shear waves by continuous acoustic vibrations with a frequency of 60 Hz. To allow image acquisition in consistent positions, the patients were asked to hold their breath after expiration. Four slices were acquired for the T1 map and the MRE sequences centered at the level of the liver hilum to cover as much liver parenchyma as possible. T1 maps and liver stiffness (LS) maps were generated by the scanner software (Siemens MapIt).

### Image analysis

**Fig. 1:**
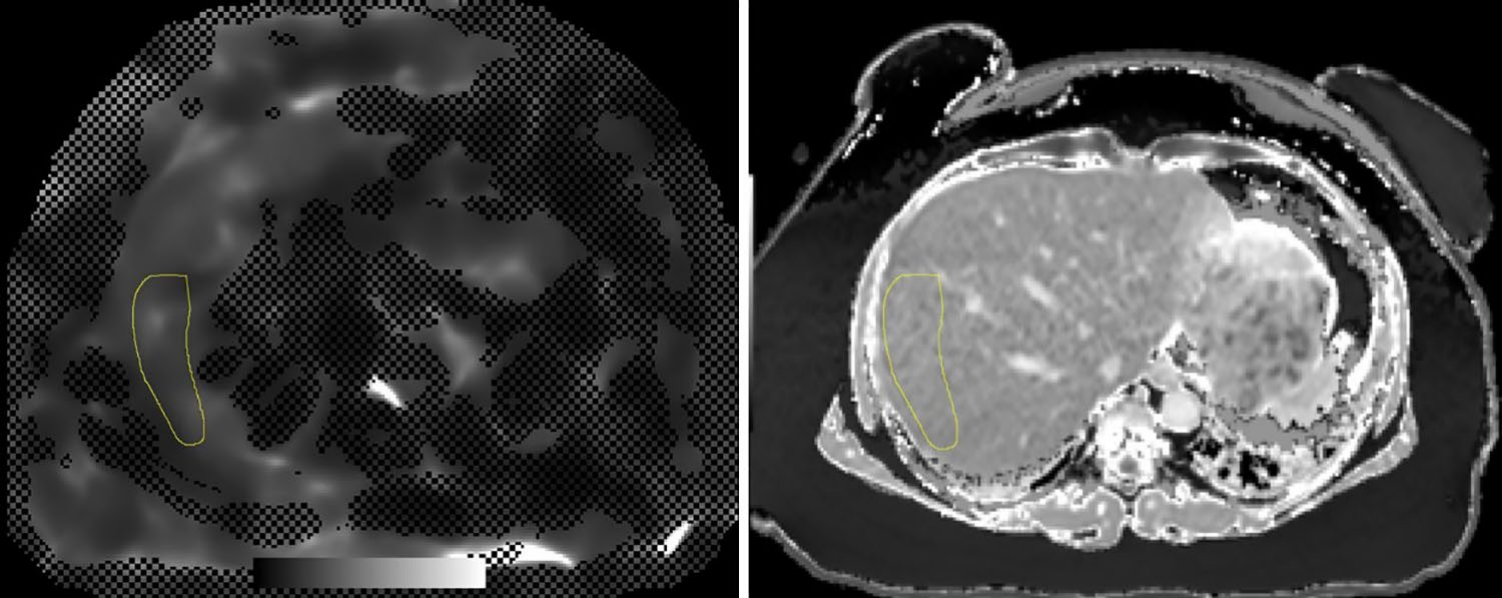
Example of the identical size and positioning of the ROI on the T1 map and MRE confidence map performed with ImageJ

Image analysis was performed by two readers independently (3 and 2 years of experience in abdominal MRI). For the measurements of T1 and LS values, the program ImageJ was used (Image J 1.52 k, Wayne Rasband National Institutes of Health, USA). Regions of interest (ROI) were drawn on MRE LS confidence maps by looking at the wave images and the anatomical information of the magnitude images: two ROIs were drawn in the right liver—one in segment V/VI and one in segment VII/VIII—whenever possible. The biggest ROI size possible was obtained avoiding large vessels. The T1 map images and MRE images were scaled to the same size to achieve the same ROI size and ROIs were copied from the MRE images to the same organ position on T1 map images with the help of the anatomical MR images (Fig. 1). The mean value and standard deviation of the ROI were determined.

### Statistical analysis

Descriptive data are given as mean ± standard deviation or as absolute or relative frequency. Continuous variables were checked for normal distribution with the Shapiro–Wilk test. Inter-reader agreement for measurements of T1 and MRE LS values was assessed using intraclass correlation coefficients (ICC) [20]. The mean of all ROIs per patient was used for statistical analysis. T1 values between fibrosis stages and inflammation activity were compared using an independent T test. The MRE LS values of different fibrosis stages and different inflammation activity grades were compared using a Mann–Whitney *U* test due to non-normal distribution of the MRE data. The correlation of T1 and MRE LS values with histopathological fibrosis stage and inflammation activity grading was tested with Spearman correlation, and the correlation between T1 values and MRE LS with Pearson correlation. Furthermore, the diagnostic performance of T1 and MRE LS for staging liver fibrosis was assessed using ROC curves and using the bivariate statistical analysis (chi-square test) [21] for the inter-method comparison of ROC curves. For identifying the optimal cut-off values regarding the diagnostic performance to distinguish different fibrosis stages, the Youden index was used.

A *p* value of 0.05 was set as statistical significance level. For all statistical analysis, SPSS 25 (IBM, Armonk, NY, USA) was utilized.

## Results

### Study population

**Fig. 2:**
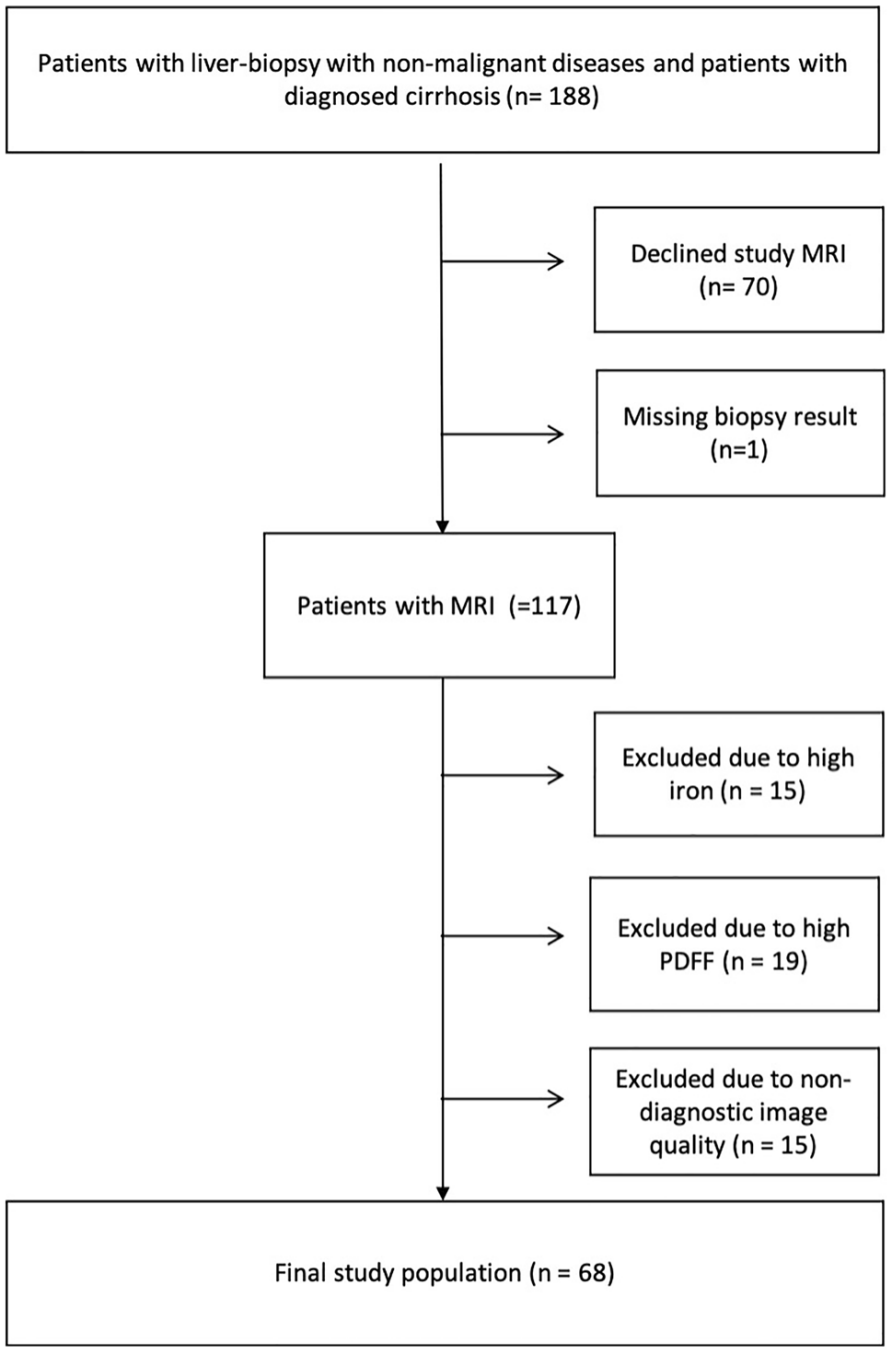
Flowchart of patient inclusion

188 patients were screened for study inclusion between August 2015 and March 2020. Out of these 188 patients, 70 patients were not willing to participate in the study. Moreover, 15 patients were excluded due to high iron load or high R2* values, 19 patients due to a PDFF > 15%, 15 patients due to non-sufficient image quality in the T1 map (*n* = 8) or MRE (*n* = 5) or no matching between MRE and T1 map (*n* = 2), and 1 patient due to a missing biopsy result. (For an overview see Fig. 2).

The final study population comprised 68 patients. Detailed patient characteristics are presented in Tables 2 and 3.

**Table 2:** Patient characteristics

Variables	Study group
Number of patients	68
Male: Female	40:28
BMI (kg/m^2^)	28.5 ± 5.7
Age (years)	48.0 ± 13.6
AST (U/l)	57.3 ± 44.4
Platelet count (G/l)	228.5 ± 81.6
Underlying liver disease (*n* = 68)	
HBV	18
NAFLD/NASH	12
ALD	9
Unknown	7
HCV	4
AIH	3
Nutritive toxic	4
ASH/NASH	2
PSC/PSC-AIH	2
Toxic/drug induced	2
PBC/PBC-AIH	2
Wilson	1
Liver donor	1
Post-liver transplantation (acute HBV)	1
Fibrosis staging (*n* = 68)	
0	9
1	21
2	11
3	10
4	17
Inflammation grade (*n* = 57)	
0	17
1	30
2	10
3	0

Values are means ± standard deviation

*BMI* Body Mass Index, *AST* Aspartate aminotransferase, *ALD* Alcoholic Liver Disease; *AIH* Autoimmune Hepatitis, *HBV* Hepatitis B Virus, *HCV* Hepatitis C Virus, *NAFLD* Non-Alcoholic Fatty Liver Disease, *NASH* Non-Alcoholic Steatohepatitis, *PBC* Primary biliary cholangitis, *PSC* Primary sclerosing cholangitis

**Table 3:** Crosstable of fibrosis stages and inflammation grade

	A0	A1	A2	Total
F0	6 (10.5%)	3 (5.3%)	0 (0%)	9 (15.8%)
F1	8 (14.0%)	9 (15.8%)	4 (7.0%)	21 (36.8%)
F2	0 (0.0%)	8 (14.0%)	3 (5.3%)	11 (19.3%)
F3	2 (3.5%)	5 (8.8%)	3 (5.3%)	10 (17.5%)
F4	1 (1.8%)	5 (8.8%)	0 (0.0%)	6 (10.5%)
Total	17 (29.8%)	30 (52.6%)	10 (17.5%)	57 (100.0%)

### T1 and MRE LS measurements for fibrosis staging

The inter-reader agreement for the two readers was excellent for measurements on T1 maps (ICC: 0.993) and MRE LS maps (ICC: 0.982). The mean size of the ROI was 343 ± 161 mm^2^.

**Fig. 3:**
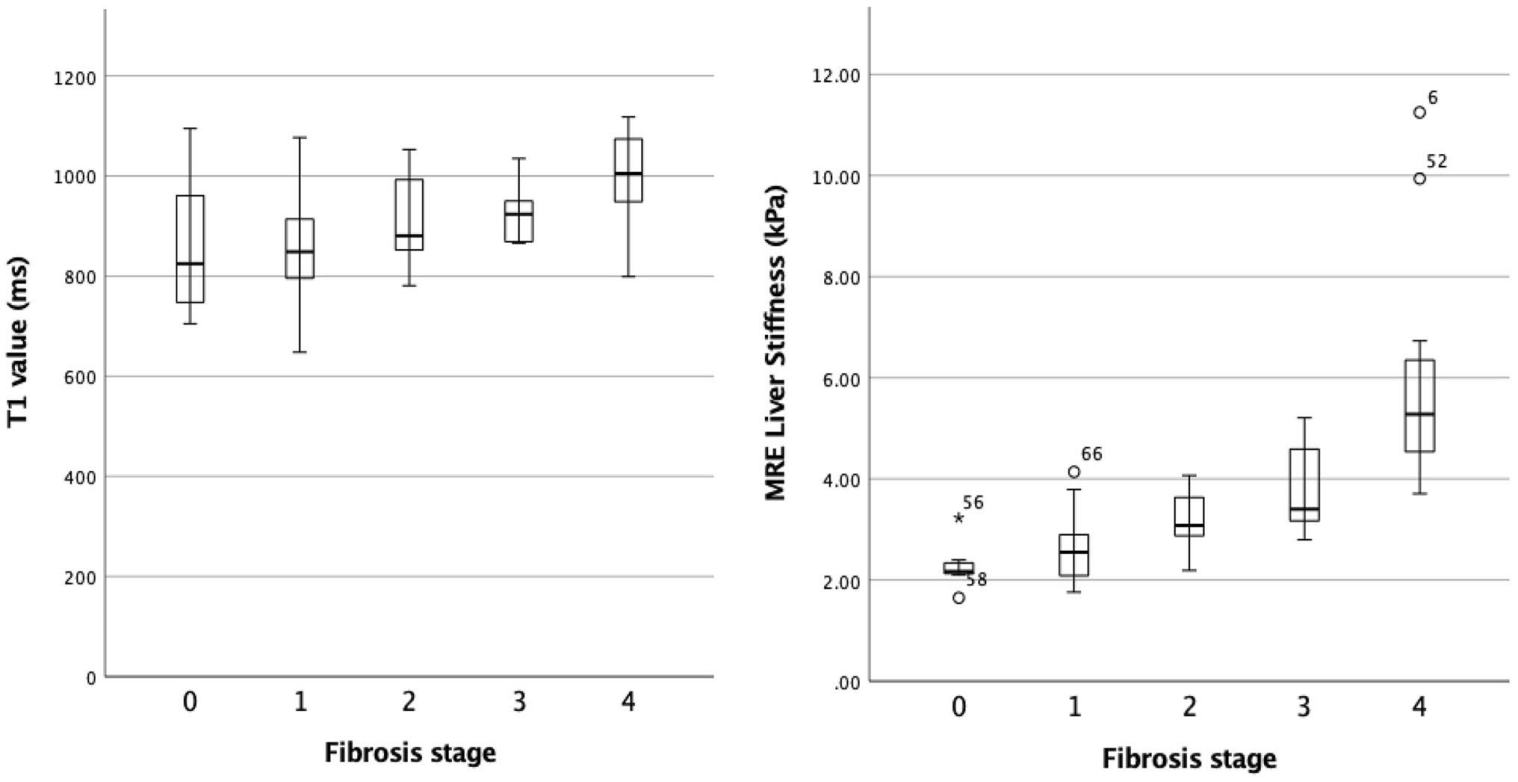
Simple Boxplot of the mean T1 values and mean MRE LS values by Fibrosis stages

**Fig. 4:**
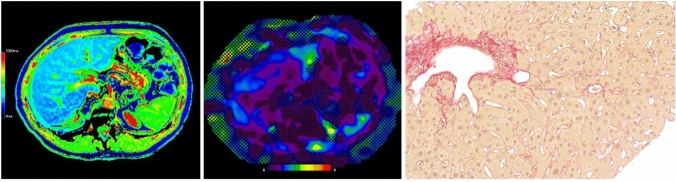
51-year-old man with low-stage fibrosis (F1) caused by an unknown liver disease. The T1 Map (left) showed a mean T1 relaxation time of 671 ms and MRE (middle) showed a mean liver stiffness value of 2.10 kPa. Correlating histology image with Sirius red staining at 300 × magnification (right)

**Fig. 5:**
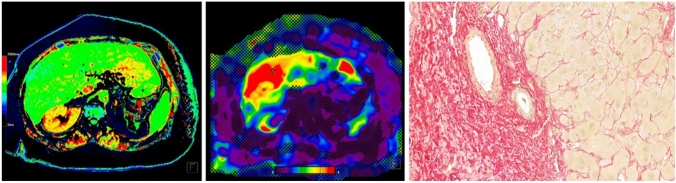
65-year-old male with cirrhosis (F4) caused by ALD. The T1 map (left) showed a mean T1 relaxation time of 1098 ms and MRE (middle) a mean stiffness value of 5.91 kPa. Correlating histology image with Sirius red staining at 300 × magnification (right)

The mean T1 values and LS values per fibrosis stage are given in Table 4 and plotted in boxplots (Fig. 3). The mean T1 values in patients with significant fibrosis (F2-4) were significantly higher compared to patients with no or low-stage fibrosis (F0-1) (955.1 ± 88.4 ms vs. 851.4 ± 103.3 ms, *p* < 0.0001). Also, for patients with severe fibrosis (F3-4) T1 values were significantly higher compared to patients without and with low or moderate fibrosis (F0-2) (971.4 ± 84.8 ms vs. 868.4 ± 102.3 ms, *p* < 0.0001) (Fig. 4 and 5).

**Table 4:** Measurements of T1 mapping and MRE by Fibrosis stage and Inflammation grade

	All (*n* = 68)	F0 (*n* = 9)	F1 (*n* = 21)	F2 (*n* = 11)	F3 (*n* = 10)	F4 (*n* = 17)
T1 value (ms)	909.3 ± 107.8	848.4 ± 135.9	852.7 ± 89.9	914.8 ± 87.6	922.5 ± 53.3	1000.2 ± 87.8
	(648–1118)	(705–1095)	(648–1077)	(781–1053)	(866–1035)	(799–1118)
MRE liver stiffness (kPa)	3.62 ± 1.75	2.28 ± 0.42	2.62 ± 0.65	3.17 ± 0.61	3.72 ± 0.81	5.80 ± 2.01
	(1.65–11.25)	(1.65–3.23)	(1.76–4.14)	(2.19–4.07)	(2.80–5.21)	(3.71–11.25)

	All (*n* = 57)	A0 (*n* = 17)	A1 (*n* = 30)	A2 (*n* = 10)	A3 (*n* = 0)	
T1 value (ms)	889.6 ± 103.5	833.5 ± 123.0	905.7 ± 92.7	927.9 ± 68.2	–	
	(648–1097)	(648–1097)	(765–1083)	(847–1053)		
MRE Liver Stiffness (kPa)	3.26 ± 1.50	2.69 ± 0.95	3.53 ± 1.86	3.41 ± 0.59		
	(1.65–11.25)	(1.65–5.67)	(1.76–11.25)	(2.55–4.59)		

Values are means ± standard deviation, range in brackets

*MRE* Magnetic resonance elastography, *F*0, *F*1, *F*2, *F*3, and *F*4: fibrosis stages, *A*0, *A*1, *A*2, and *A*3: inflammation activity grading

The mean LS was significantly higher in patients with significant fibrosis (F2-4) compared to patients with no or low-stage fibrosis (F0-1) (4.49 ± 1.87 kPa vs. 2.52 ± 0.60 kPa, *p* < 0.0001) and between patients with severe fibrosis (F3-4) compared to patients without and with low or moderate fibrosis (F0-2) (5.03 ± 1.94 kPa vs. 2.70 ± 0.67 kPa, *p* < 0.0001) (Figs. 4 and 5).

T1 values showed a moderate (*r* = 0.547, *p* < 0.0001) and MRE LS values a good correlation (*r* = 0.813, *p* < 0.0001) with histopathologic fibrosis stage, respectively. T1 values positively correlated with MRE LS values (*r* = 0.533, *p* < 0.0001).

### T1 and MRE LS measurements for inflammation grading

**Fig. 6:**
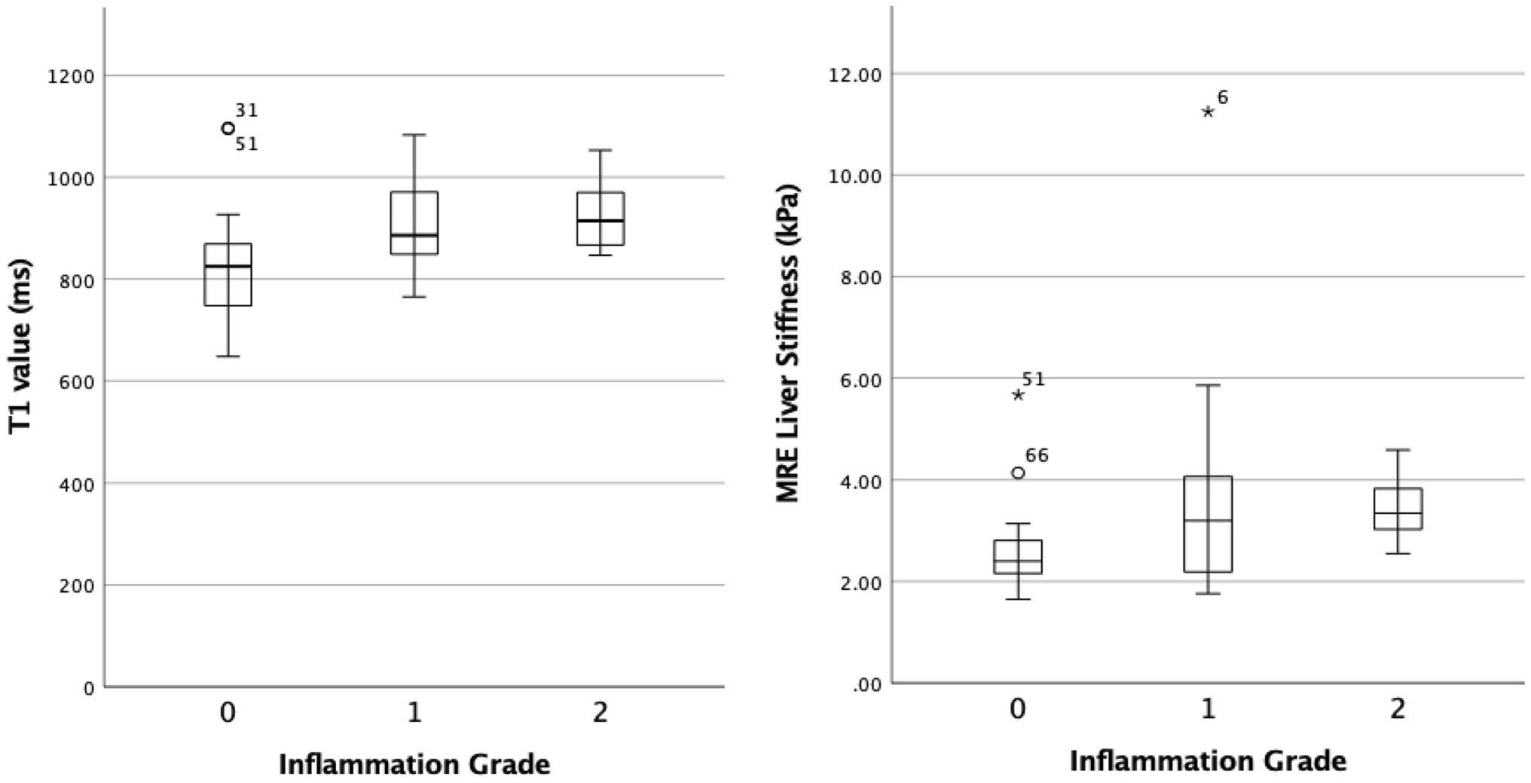
Simple Boxplot of the mean T1 values and mean MRE LS values by Inflammation grade

The mean T1 values and LS values per inflammation grade are given in Table 4 and plotted in boxplots (Fig. 6). There was no significant difference between the mean T1 values in patients with no or low inflammation (A0-1) and patients with moderate inflammation (A2) (881.14 ± 108.4 ms vs. 935.7 ± 68.7 ms, *p* = 0.20). However, there was a significant difference of the mean T1 values in patients with no inflammation (A0) and low to moderate inflammation (A1-2) (838.5 ± 123.0 ms vs. 911.3 ± 86.9 ms, *p* = 0.01).

The mean LS likewise showed no significant difference in patients with no or low inflammation (A0-1) and patients with moderate inflammation (A2) (3.23 ± 1.63 kPa vs. 3.41 ± 0.59 kPa, *p* = 0.10) and a significant difference between patients with no inflammation (A0) and low to moderate inflammation (A1-2) (2.69 ± 0.95 vs. 3.50 ± 1.63 kPa, *p* = 0.01).

T1 values (*r* = 0.352, *p* = 0.01) and MRE LS values (*r* = 0.355, *p* = 0.01) showed a low correlation with histopathologic inflammation activity grades.

### Diagnostic performance of T1 mapping and MRE

**Fig. 7:**
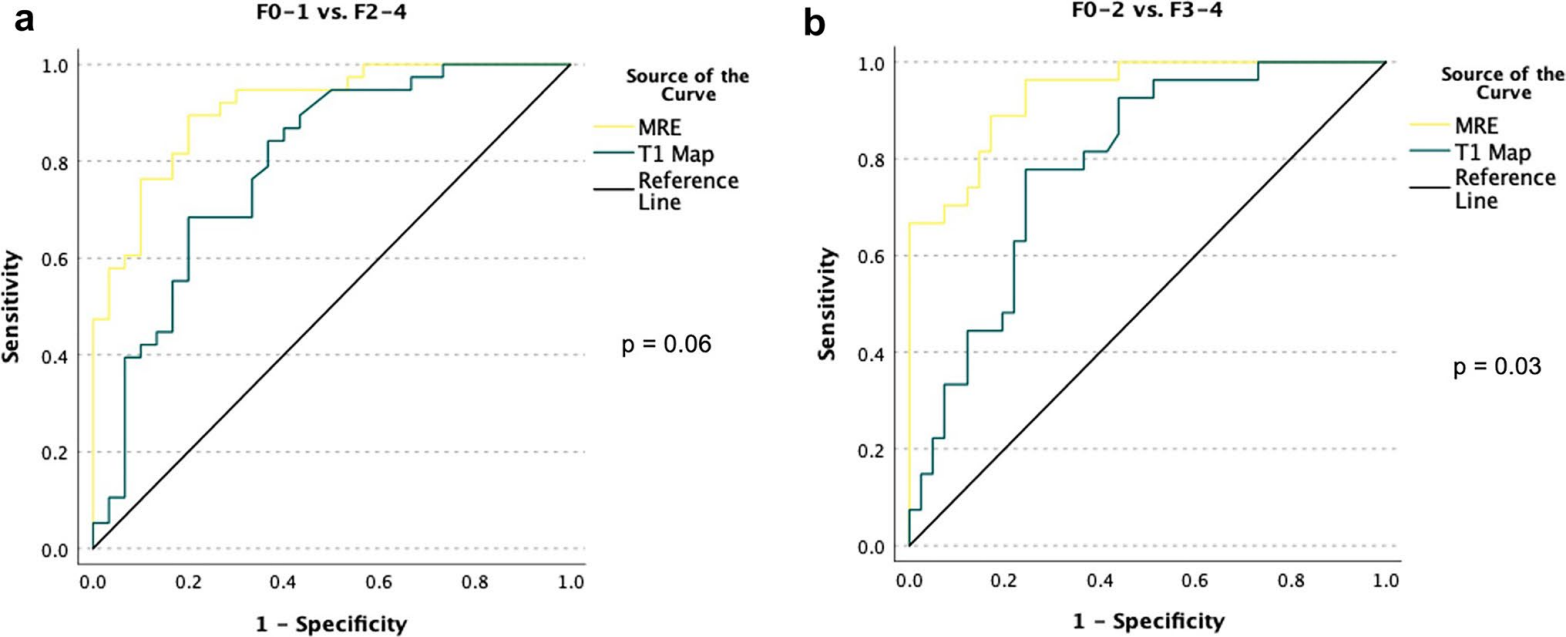
**a** ROC of the diagnostic performance of MRE (yellow line) and T1 map (green line) for fibrosis stages. The AUC showed similar results in distinguishing no or low fibrosis stages (F0-1) from significant fibrosis stages (F2-4): AUC value T1 map 0.79 vs. AUC value MRE 0.91 (*p* = 0.06). **b** ROC of the diagnostic performance of MRE (yellow line) and T1 map (green line) for fibrosis stages. The AUC showed significantly lower detection of severe fibrosis (F3-4) for T1 mapping: AUC value T1 map 0.79 vs. AUC value MRE 0.94 (*p* = 0.03)

The ROC analysis revealed a lower AUC for T1 values than MRE LS for diagnosing significant fibrosis (F2-F4) (AUC 0.79, 95% confidence interval (CI): 0.68–0.90 vs. 0.91; 95% CI 0.84–0.98, *p* = 0.06) without a significant difference. However, T1 mapping showed a significantly lower diagnostic performance than MRE LS for severe fibrosis (F3-4; AUC 0.79, 95% CI 0.68–0.90 vs. 0.94, 95% CI 0.88–0.99 *p* = 0.03) (Fig. 7a and b).

The optimal cut-off values for MRE LS were 2.42 kPa for F1-4, 2.92 kPa for F2-4, 3.13 kPa for F3-4, and 3.65 kPa for F4. For T1 values, the cut-off values were 845.5 ms for F1-4, 918.0 ms for F2-4, 920 ms for F3-4, and 940 ms for F4 (Table 5).

**Table 5:** Cut-off Values of T1 values and MRE LS for different fibrosis stages

T1 values	≥ F1	≥ F2	≥ F3	F4
Cut-off values (ms)	845.5	918.0	920.0	940.0
Sensitivity	81%	68%	78%	82%
Specificity	67%	80%	76%	76%

MRE LS	≥ F1	≥ F2	≥ F3	F4
Cut-off values (kPa)	2.42	2.92	3.13	3.65
Sensitivity	81%	90%	96%	100%
Specificity	89%	80%	76%	84%

**Fig. 8:**
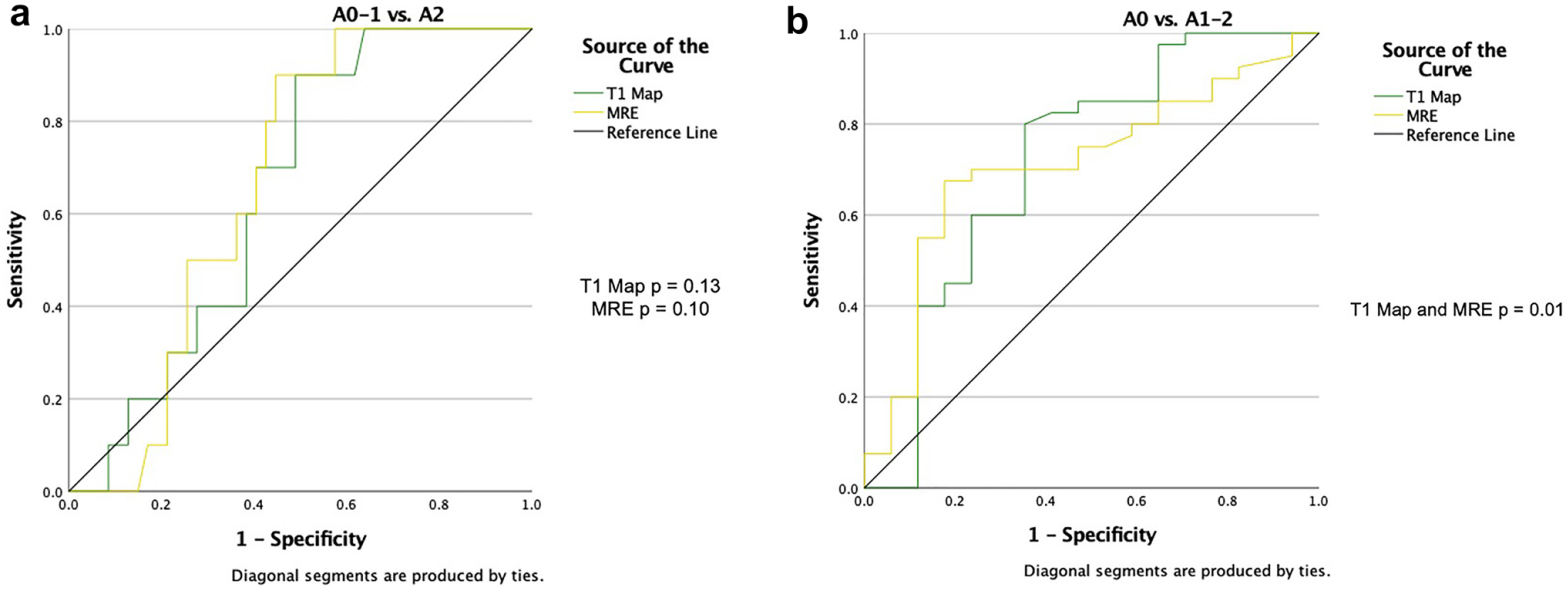
**a** ROC of the diagnostic performance of MRE (yellow line) and T1 map (green line) for inflammation activity grading. The AUC showed a low, non-significant performance in the detection of moderate inflammation (A2) for both MRE (AUC 0.67, *p* = 0.10) and T1 map (AUC 0.65, *p* = 0.13). **b** ROC of the diagnostic performance of MRE (yellow line) and T1 map (green line) for inflammation activity grading. The AUC showed a low, but significant performance in the detection of low to moderate inflammation (A1-2) for both MRE (AUC 0.71, *p* = 0.01) and T1 map (AUC 0.72, *p* = 0.01)

For the diagnosis of moderate inflammation, the ROC analysis showed a low AUC for both T1 values (AUC 0.65, 95% CI 0.51–0.80, *p* = 0.13) and MRE LS (AUC 0.67, 95% CI 0.51–0.80, *p* = 0.10) without statistical significance (Fig. 8a). The diagnosis of low to moderate inflammation revealed a better and similar AUC for both T1 values (AUC 0.72, 95% CI 0.55–0.88, *p* = 0.01) and MRE LS (AUC 0.71, 95% CI 0.57–0.86, *p* = 0.01) (Fig. 8b).

## Discussion

Our study shows that Look-Locker T1 mapping can identify significant and severe hepatic fibrosis as diagnosed by liver biopsy in patients with chronic liver disease. T1 values increased with hepatic fibrosis stages and showed a moderate and significant correlation with hepatic fibrosis stages. However, the diagnostic performance of T1 mapping in diagnosing severe fibrosis is significantly lower than that of MRE. Regarding hepatic inflammation both T1 mapping and MRE showed only a low correlation with inflammation activity grades and a lower diagnostic performance than for fibrosis staging.

Similar to our results, two animal studies by Li et al. [11] measuring T1 relaxation time in rabbits with induced liver fibrosis and Chow et al. [22] using a mouse model of induced liver fibrosis showed an increase of the T1 relaxation time in animals with increasing liver fibrosis. In patient studies T1 relaxation time was significantly increased in liver cirrhosis [23] and gradually increased with increasing fibrosis stage based on liver biopsy results [12, 16].

In our study, the T1 relaxation times in patients without fibrosis (856.3 ± 128.7 ms) are within the range of published numbers. Reported T1 values of normal liver at 3.0 Tesla ranged between 809 ± 71 ms and 941 ± 136 ms [13, 24, 25].

With T1 mapping, we were able to diagnose patients with clinically significant fibrosis (≥ F2), which would prompt further treatment. T1 values might not only correlate with clinically significant fibrosis but also predict clinical outcome in patients with hepatic fibrosis [26]. Furthermore, T1 values could serve as biomarker for treatment response assessment as shown in a recent study, where T1 values significantly decreased following antiviral therapy in patients with chronic hepatitis C [27]. The diagnostic performance of T1 mapping for detecting severe fibrosis was 0.79 (95% confidence interval (CI) 0.68–0.90) in our study, which is comparable to a previously demonstrated AUROC of 0.81 (95% CI 0.65–0.96) [16] in a mixed study population. In a study with NAFLD patients, the reported AUC was slightly higher (0.837) for detecting significant fibrosis [28]. The diagnostic performance of T1 mapping lies between other methods for assessing significant and severe fibrosis, for example: 0.63–0.67 for F2-4 and 0.59–0.72 for F3-4 for apparent diffusion coefficient (ADC) [29, 30], 0.79–0.89 for F2-4 and 0.83–0.92 for F3-4 for vibration-controlled transient elastography (VCTE) [31, 32], and 0.80–0.87 for F2-4 and 0.80–0.90 for F3-4 for two-dimensional shear wave elastography (2D-SWE) [33–35]. Even though T1 mapping demonstrates a diagnostic performance a little below ultrasound-based methods, it comes with the advantage of high robustness in terms of inter-reader agreement as seen in our study.

Comparing the diagnostic performance of T1 mapping and MRE, we found a significantly higher AUC for detecting F3-4 fibrosis with MRE (AUC = 0.94). MRE also showed a high performance in detecting the other fibrosis stages (AUC F2-4 = 0.91, AUC F4 = 0.97). These results are in line with previous studies, as for example, in Chen et al. (AUC F2-4 = 0.93, AUC F3-4 = 0.92, and AUC F4 = 0.95) [36] and Lefebvre et al. (AUC F2-4 = 0.85, AUC F3-4 = 0.88, and AUC F4 = 0.88) [37]. Also the LS values in our study are comparable to previous studies at 3.0 Tesla for normal liver with 2.3 ± 0.4 kPa (between 2.14 ± 0.33 [38] and 2.6 ± 0.4 kPa [39]) and per fibrosis stage with 2.6 ± 0.7 kPa for F1, 3.2 ± 0.6 kPa for F2, 3.7 ± 0.8 kPa for F3, and 5.8 ± 2.0 kPa for F4 (F1 2.4–3.4 kPa, F2 2.7–3.8 kPa, F3 3.3–6.0 kPa, and F4 4.4–8.2 kPa [37, 39, 40]).

Two previous studies showed a positive, but low correlation of T1 relaxation time of the liver with LS measured by MRE serving as a surrogate for hepatic fibrosis (*r* = 0.36–0.49, *p* < 0.001) [14, 41]. Moreover, a recent study also showed a good diagnostic performance for T1 mapping in detecting liver cirrhosis, however only with serum markers and MRE as reference of standard [42]. We were not only able to reproduce these findings but also to directly show the positive correlation of T1 relaxation time with histopathological assessed hepatic fibrosis stage. Hence, T1 mapping as shown in our study might be an alternative to MRE, especially in patients with ascites, intrathoracic liver position, and high body mass index or when MRE is not available [17]. Nonetheless, T1 mapping proofed to be not as accurate in our study as MRE in detecting significant and severe fibrosis, therefore whenever MRE is available and the patient is suitable for MRE, MRE should remain the standard for assessing liver fibrosis. The higher diagnostic accuracy of MRE in comparison to T1 mapping is supported by other recent study results [14, 28]. The lower diagnostic performance of T1 mapping in our study may be explained by the overlap of T1 values especially between lower stages of fibrosis. Similar overlaps in T1 values between patients without and with advanced hepatic fibrosis have been reported recently [13, 14].

Regarding the assessment of hepatic inflammation Hoad et al. [16] showed that T1 values of the liver were significantly increased in patients with moderate to severe hepatic inflammation in a group without or low-stage fibrosis, but not in the group with advanced fibrosis. In a study by Kim JW et al. [28] in a cohort of NAFLD patients the AUC for diagnosing inflammation activity (ballooning ≥ B1 and ≥ B2, lobular inflammation ≥ L2) was 0.624–0.686 for T1 mapping and 0.765–0.898 for MRE. Differences in AUCs to our results may be explained by a different histopathological scoring system used for activity grading and the higher number of patients with high-grade activity compared to our study.

Potential confounders in T1 mapping are hepatic iron overload with a decrease of T1 relaxation time [16] and hepatic steatosis with an increase of T1 relaxation time [43]. The Look-Locker technique, however, is less influenced by hepatic iron deposition than other T1 mapping techniques. [44]. Hence, to broaden the spectrum of patients potentially benefitting from this non-invasive hepatic fibrosis assessment, we recommend a multiparametric MR-approach comprising not only T1 mapping, but also methods to quantify hepatic steatosis (namely PDFF measurements) and iron in order to appraise other liver conditions that can influence the non-invasive grading of fibrosis and inflammation [12].

This study has several limitations. First, we did not evaluate the full spectrum of chronic liver disease, which would also include patients with high iron and fat deposition in the liver. However, we were able to demonstrate a good diagnostic performance of T1 mapping and MRE even in patients with low stages of hepatic fibrosis. This can now lay the ground for further studies with a multiparametric approach to assess chronic liver disease to overcome this limitation. Second, the assessment of hepatic inflammation did not include patients with severe inflammation, which is due to the inclusion criteria of the study, where patients with chronic and not acute liver disease were included.

Fourth, the study population was small and therefore patients with very high values in the MRE or T1 map might distort the actual performance of these tools. Hence, we recommend further evaluation in larger patient samples.

In conclusion, T1 mapping can diagnose significant and severe hepatic fibrosis, however with a tendency toward lower diagnostic performance compared to MRE. Thus, whenever MRE is not available or likely to fail due to intrinsic factors of the patient, T1 mapping may be used as an alternative to MRE for diagnosing hepatic fibrosis. Both T1 mapping and MRE are probably not sufficient as standalone methods to diagnose hepatic inflammation with relatively low diagnostic accuracy.

Further studies are needed to overcome the limitations of T1 mapping in patients with hepatic iron deposition and steatosis and to further elucidate the role of T1 mapping in diagnosing hepatic inflammation.
